# Discussion With Providers of Memory Difficulties Among Adults 45+: Demographic Differences

**DOI:** 10.1093/geroni/igaf122.2453

**Published:** 2025-12-31

**Authors:** Drew Blasco, Jace Flatt

**Affiliations:** University of Nevada, Las Vegas, Las Vegas, Nevada, United States; University of Nevada, Las Vegas, Las Vegas, Nevada, United States

## Abstract

Subjective cognitive decline (SCD), may indicate increased risk for Alzheimer’s disease—a critical area for intervention given the aging population. 2023 Behavioral Risk Factor Surveillance System data (optional SCD and Social Determinants of Health (SDoH) modules administered in 20 states and 1 territory) was utilized to understand the association between participant demographics (age, sex, race and ethnicity, education, income, health insurance, and social determinants of health) and whether participants with SCD/anyone else discussed difficulties with thinking/memory with a healthcare provider. The sample (n = 14,254) was on average 66 years old (range:45-80), majority female (∼58%) and insured (∼97%), with almost 25% identifying as a person of color and about 42% reporting poor/fair health. Bivariate associations demonstrated differences with those of slightly younger age, female (45% vs 38%), insured (44% vs 25%), and reporting lower incomes more likely to have discussed SCD with a provider while those who identified as Asian Americans (30%) and Native Hawaiian and other Pacific Islander (34%) compared to White (44%) and Hispanic/Latino (49%) and those with less education were less likely to have discussed with a provider. Similar patterns were found for results of the multivariable logistic regression analysis including significant demographic factors of age, sex, education, health insurance, and race and ethnicity when examining the likelihood of talking to a provider. Discussion of subjective memory concerns with a healthcare professional may aid in improving the health of middle-to-older age adults; however, important to consider are potential barriers to provider discussion which may increase health disparities.

